# Relationship between COVID-19 perceived risk and perfectionism – a preliminary study

**DOI:** 10.1192/j.eurpsy.2021.827

**Published:** 2021-08-13

**Authors:** A.T. Pereira, C. Cabaços, T. Soares, A. Araujo, R. Sousa, A. Macedo

**Affiliations:** 1 Institute Of Psychological Medicine, Faculty of Medicine, University of Coimbra, Coimbra, Portugal; 2 Institute Of Psychological Medicine, Faculty Of Medicine, University of Coimbra, coimbra, Portugal; 3 Usf Coimbra Centro, USF Coimbra Centro, Coimbra, Portugal

**Keywords:** COVID-19, Covid-19 Perceived Risk, Perfectionism, psychological impact

## Abstract

**Introduction:**

Research following the Covid-19 pandemics has shown that psychological reactions to the pandemic and its constraints can vary significantly depending on personality. One of the traits that has not been studied yet, but can play a harmful role in the COVID-19 psychological impact is perfectionism. This trait, characterized by setting excessively high standards of performance and striving for flawlessness, has increased in recent years and is considered a transdiagnostic process involved in several (mental) health problems (Curran & Hill 2019).

**Objectives:**

To analyze the role of Perfectionism in the levels of fear of COVID19 and of perception of infection risk by COVID-19.

**Methods:**

234 adults (75.6% women; mean age=29.53±12.51) completed an on-line survey with the Portuguese validated versions of Covid-19 Perceived Risk Scale (C19PRS; Pereira et al. 2020), Fear of COVID-19 Scale (FC19S; Cabaços et al. 2020) and Big Three Perfectionism Scale (BTPS; Garrido et al. 2020). SPSS was used to perform correlation and regression analysis.

**Results:**

Perceived Risk and Fear of COVID-19 were significantly correlated with perfectionism (.243, .228, respectively) (both, p<.01). Perfectionism explains 5.5% (Adjusted R2) of the FC19S variance (Beta=.243, p<.001) and 4.8% of the C19PRS variance (Beta=.228, p=.01).

**Conclusions:**

This study provides preliminary, but completely innovative evidence that perfectionism contribute to the psychological impact of Covid-19 pandemics. In the near future we will test the hypothesis that the nature of unpredictability and the limitations imposed by the global crisis may be exacerbating the already high levels of psychological distress that affect negative perfectionists.

